# Hexa­aqua­nickel(II) di­hydrogen hypodiphosphate

**DOI:** 10.1107/S1600536813030717

**Published:** 2013-11-13

**Authors:** Mimoza Gjikaj, Peng Wu, Niels-Patrick Pook

**Affiliations:** a Institute of Inorganic and Analytical Chemistry, Clausthal University of Technology, Paul-Ernst-Strasse 4, D-38678 Clausthal-Zellerfeld, Germany

## Abstract

The asymmetric unit of the title compound, [Ni(H_2_O)_6_](H_2_P_2_O_6_), contains one-half of the hexa­aqua­nickel(II) cation and one-half of the di­hydrogen hypodiphosphate anion. In the complex cation, the Ni^2+^ atom is located on an inversion center and has an octa­hedral coordination sphere. The P—P distance in the centrosymmetric anion is 2.1853 (7) Å. In the crystal, discrete [Ni(H_2_O)_6_]^2+^ cations and (H_2_P_2_O_6_)^2−^ anions are stacked in columns parallel to the *c* axis and are linked into a three-dimensional network by medium-strength O—H⋯O hydrogen bonds.

## Related literature

For the synthesis of hypodiphosphates, see: Leininger & Chulski (1953 ▶). For applications of hypodiphosphates, see: Bloss & Henzel (1967 ▶); Ruflin *et al.* (2007 ▶); Szklarz *et al.* (2011 ▶). For the crystal structures of transition metal hypodiphosphate 12-hydrates, see: Hagen & Jansen (1995 ▶); Haag *et al.* (2005 ▶). For the crystal structures of hydrogen hypodiphosphate compounds, see: Collin & Willis (1971 ▶); Szafranowska *et al.* (2012 ▶); Wu *et al.* (2012 ▶).

## Experimental

### 

#### Crystal data

[Ni(H_2_O)_6_](H_2_P_2_O_6_)
*M* *_r_* = 326.76
Monoclinic, 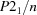

*a* = 9.3031 (14) Å
*b* = 5.8892 (8) Å
*c* = 9.7620 (12) Å
β = 96.111 (11)°
*V* = 531.80 (13) Å^3^
*Z* = 2
Mo *K*α radiation
μ = 2.18 mm^−1^
*T* = 223 K
0.29 × 0.25 × 0.22 mm

#### Data collection

Stoe IPDS-II diffractometer
Absorption correction: numerical (*X-SHAPE* and *X-RED*; Stoe & Cie, 1999 ▶, 2001 ▶) *T*_min_ = 0.537, *T*_max_ = 0.619
8061 measured reflections
1601 independent reflections
1546 reflections with *I* > 2σ(*I*)
*R*_int_ = 0.056

#### Refinement

*R*[*F* ^2^ > 2σ(*F* ^2^)] = 0.029
*wR*(*F* ^2^) = 0.074
*S* = 1.16
1601 reflections
98 parameters
All H-atom parameters refined
Δρ_max_ = 0.44 e Å^−3^
Δρ_min_ = −1.08 e Å^−3^

### 

Data collection: *X-AREA* (Stoe & Cie, 2002 ▶); cell refinement: *X-AREA*; data reduction: *X-AREA*; program(s) used to solve structure: *SHELXS97* (Sheldrick, 2008 ▶); program(s) used to refine structure: *SHELXL97* (Sheldrick, 2008 ▶); molecular graphics: *DIAMOND* (Brandenburg, 2012 ▶); software used to prepare material for publication: *SHELXL97*, *PLATON* (Spek, 2009 ▶) and *publCIF* (Westrip, 2010 ▶).

## Supplementary Material

Crystal structure: contains datablock(s) I. DOI: 10.1107/S1600536813030717/wm2784sup1.cif

Structure factors: contains datablock(s) I. DOI: 10.1107/S1600536813030717/wm2784Isup2.hkl

Additional supplementary materials:  crystallographic information; 3D view; checkCIF report

## Figures and Tables

**Table 1:** Hydrogen-bond geometry (Å, °)

*D*—H⋯*A*	*D*—H	H⋯*A*	*D*⋯*A*	*D*—H⋯*A*
O3—H3⋯O2^i^	0.92 (3)	1.68 (3)	2.5919 (15)	168 (3)
O4—H4*A*⋯O3^ii^	0.82 (3)	1.93 (3)	2.7293 (16)	165 (3)
O4—H4*B*⋯O2^iii^	0.82 (3)	1.89 (3)	2.6822 (14)	162 (3)
O5—H5*A*⋯O1	0.81 (3)	1.98 (3)	2.7770 (15)	171 (3)
O5—H5*B*⋯O2^iv^	0.84 (3)	2.04 (3)	2.8722 (16)	171 (3)
O6—H6*A*⋯O1^v^	0.81 (2)	2.05 (2)	2.8163 (16)	159 (2)
O6—H6*B*⋯O1^ii^	0.84 (3)	2.08 (3)	2.9132 (17)	168 (3)

Symmetry codes: (i) 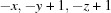; (ii) 
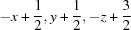
; (iii) 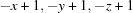; (iv) 
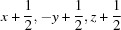
; (v) 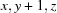.

**Fig. 1.**
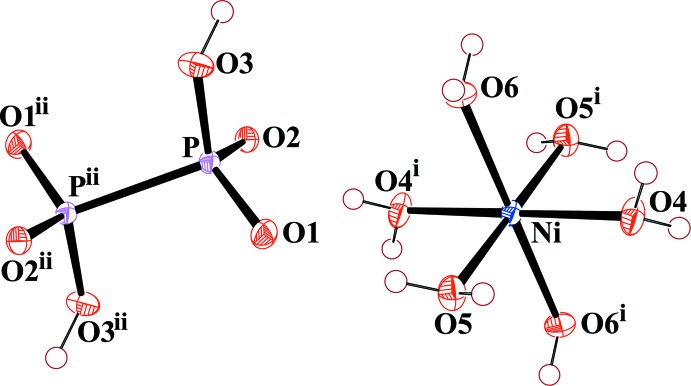
The molecular entities in the title compound with atom labels and displacement ellipsoids drawn at the 50% probability level. [Symmetry codes: (i) -x + 1, -y + 1, -z + 1; (ii) -x, -y, -z + 1.]

**Fig. 2.**
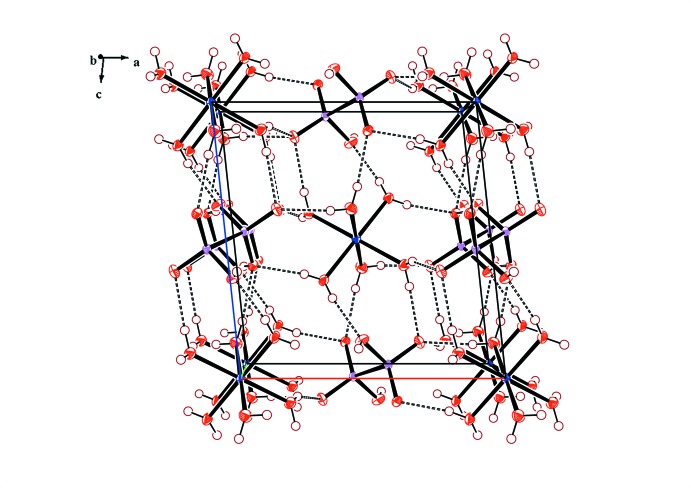
The crystal structure of the title compound viewed along the b axis. Displacement ellipsoids are drawn at the 50% probability level. O—H···O hydrogen bonds are shown as dashed lines.

## supplementary crystallographic information

### 1. Comment

Although main group hypodiphosphates have rather academic inter­est, the di­hydrogen sodium and ammonium derivates, (*M*_2_H_2_P_2_O_6_ with *M* = Na^+^ and NH_4_^+^) (Collin & Willis, 1971), respective their hydrates, have certain practical use. Ammonium di­hydrogen hypodiphosphate-based powder material is applied as a green flame retardant and as a multipurpose extinguishing agent suitable for all classes of fire (Ruflin *et al.*, 2007). Acidic Na_2_H_2_P_2_O_6_ solutions are applied for gravimetric precipitation and immobilization of uranium(IV) (Bloss & Henzel, 1967). The ferroelectricity of di­ammonium hypodiphosphate was discovered recently (Szklarz *et al.*, 2011).

The crystal structures of transition metal hypodiphosphate 12-hydrates *M*_2_P_2_O_6_^.^12H_2_O (*M* = Co und Ni) were determined by Hagen & Jansen (1995) and Haag *et al.* (2005). However, the crystal structures of transition metal di­hydrogen hypodiphosphate hydrates have not been reported up to now.

The structure of the title compound, [Ni(H_2_O)_6_](H_2_P_2_O_6_) is characterized by discrete [Ni(H_2_O)_6_]^2+^ cations and (H_2_P_2_O_6_)^2-^ anions (Fig. 1). The Ni^2+^ ion is located on an inversion centre and is o­cta­hedrally coordinated by water molecules. In the (H_2_P_2_O_6_)^2-^ anion, which is located about an inversion centre, the tetra­valent phospho­rus atom is surrounded by three oxygen atoms and one additional phospho­rus atom with a P–P distance of 2.1854 (7) Å. The P–O bond lengths are ranging from 1.5050 (11) to 1.5816 (11) Å. All bond lengths and angles are well within the expected ranges (Wu *et al.*, 2012) and are comparable to those found for Ni_2_P_2_O_6_^.^ 12H_2_O (2.1700 (13) Å for the P—P bond; Haag *et al.*, 2005), however with a characteristically longer P—OH distance in comparison with the P—O distance.

The (H_2_P_2_O_6_)^2–^ anions are joined to ribbons parallel to [010] by rather strong hydrogen bonds (O···O 2.5919 (15) Å). Anions and complex cations are stacked alternately in columns parallel to [001], held together by medium-strength O—H···O hydrogen bonds (Fig. 2). The O···O distances between water molecules and (H_2_P_2_O_6_)^2–^ ions range from 2.6822 (14) to 2.9132 (17) Å (Table 1). All hydrogen bonding inter­actions considered, a three-dimensional network structure is established in the crystal.

### 2. Experimental

Disodium di­hydrogen hypodiphosphate was prepared using the procedure reported by Leininger & Chulski (1953). An aqueous solution of hypodi­phospho­ric acid was obtained by passing a saturated solution of disodium di­hydrogen hypodiphosphate through a cation exchange resin (Dowex 50WX2 50-100). Single crystals of hexa­aqua­nickel(II) di­hydrogenhypodiphosphate were prepared by adding nickel hydroxide (148 mg) to a solution of hypodi­phospho­ric acid (40 mL).

### 2.1. Refinement

All hydrogen atoms were located in a difference Fourier map and were refined isotropically with no restraints.

### Crystal data

**Table:**

[Ni(H_2_O)_6_](H_2_P_2_O_6_)	*F*(000) = 336
*M**_r_* = 326.76	block, green
Monoclinic, *P*2_1_/*n*	*D*_x_ = 2.041 Mg m^−^^3^
Hall symbol: -P 2yn	Mo *K*α radiation, λ = 0.71073 Å
*a* = 9.3031 (14) Å	Cell parameters from 8465 reflections
*b* = 5.8892 (8) Å	θ = 2.9–30.5°
*c* = 9.7620 (12) Å	µ = 2.18 mm^−^^1^
β = 96.111 (11)°	*T* = 223 K
*V* = 531.80 (13) Å^3^	Block, green
*Z* = 2	0.29 × 0.25 × 0.22 mm

### Data collection

**Table:**

Stoe IPDS-II diffractometer	1601 independent reflections
Radiation source: fine-focus sealed tube	1546 reflections with *I* > 2σ(*I*)
Graphite monochromator	*R*_int_ = 0.056
ω–scans	θ_max_ = 30.5°, θ_min_ = 2.9°
Absorption correction: numerical (*X-SHAPE* and *X-RED*; Stoe & Cie, 1999, 2001)	*h* = −13→13
*T*_min_ = 0.537, *T*_max_ = 0.619	*k* = −8→8
8061 measured reflections	*l* = −13→13

### Refinement

**Table:**

Refinement on *F*^2^	Primary atom site location: structure-invariant direct methods
Least-squares matrix: full	Secondary atom site location: difference Fourier map
*R*[*F*^2^ > 2σ(*F*^2^)] = 0.029	Hydrogen site location: inferred from neighbouring sites
*wR*(*F*^2^) = 0.074	All H-atom parameters refined
*S* = 1.16	*w* = 1/[σ^2^(*F*_o_^2^) + (0.0423*P*)^2^ + 0.1511*P*] where *P* = (*F*_o_^2^ + 2*F*_c_^2^)/3
1601 reflections	(Δ/σ)_max_ = 0.001
98 parameters	Δρ_max_ = 0.44 e Å^−^^3^
0 restraints	Δρ_min_ = −1.08 e Å^−^^3^

### Special details

**Table:**

Geometry. All e.s.d.'s (except the e.s.d. in the dihedral angle between two l.s. planes) are estimated using the full covariance matrix. The cell e.s.d.'s are taken into account individually in the estimation of e.s.d.'s in distances, angles and torsion angles; correlations between e.s.d.'s in cell parameters are only used when they are defined by crystal symmetry. An approximate (isotropic) treatment of cell e.s.d.'s is used for estimating e.s.d.'s involving l.s. planes.
Refinement. Refinement of *F*^2^ against ALL reflections. The weighted *R*-factor *wR* and goodness of fit *S* are based on *F*^2^, conventional *R*-factors *R* are based on *F*, with *F* set to zero for negative *F*^2^. The threshold expression of *F*^2^ > σ(*F*^2^) is used only for calculating *R*-factors(gt) *etc*. and is not relevant to the choice of reflections for refinement. *R*-factors based on *F*^2^ are statistically about twice as large as those based on *F*, and *R*- factors based on ALL data will be even larger.

### Fractional atomic coordinates and isotropic or equivalent isotropic displacement parameters (Å^2^)

**Table:**

	*x*	*y*	*z*	*U*_iso_*/*U*_eq_	
Ni	0.5000	0.5000	0.5000	0.01306 (10)	
P	0.06859 (3)	0.14612 (5)	0.53007 (3)	0.01116 (10)	
O1	0.20361 (11)	0.05887 (19)	0.61176 (12)	0.0177 (2)	
O2	0.08555 (10)	0.28724 (17)	0.40289 (10)	0.01404 (19)	
O3	−0.02399 (12)	0.28534 (18)	0.62787 (11)	0.0185 (2)	
O4	0.64678 (11)	0.6071 (2)	0.65421 (12)	0.0211 (2)	
O5	0.48685 (12)	0.20885 (19)	0.61392 (12)	0.0204 (2)	
O6	0.32539 (11)	0.62296 (19)	0.59655 (12)	0.0180 (2)	
H3	−0.042 (3)	0.436 (5)	0.605 (3)	0.037 (7)*	
H4A	0.620 (3)	0.681 (5)	0.719 (3)	0.037 (7)*	
H4B	0.720 (3)	0.664 (5)	0.630 (3)	0.042 (7)*	
H5A	0.407 (4)	0.154 (5)	0.607 (3)	0.045 (8)*	
H5B	0.521 (3)	0.223 (5)	0.696 (3)	0.039 (7)*	
H6A	0.296 (2)	0.750 (4)	0.581 (2)	0.019 (5)*	
H6B	0.313 (3)	0.584 (5)	0.678 (3)	0.036 (7)*	

### Atomic displacement parameters (Å^2^)

**Table:**

	*U*^11^	*U*^22^	*U*^33^	*U*^12^	*U*^13^	*U*^23^
Ni	0.01080 (13)	0.01511 (15)	0.01334 (15)	−0.00152 (7)	0.00159 (9)	−0.00086 (8)
P	0.00991 (15)	0.01073 (17)	0.01287 (17)	−0.00066 (9)	0.00128 (11)	0.00094 (10)
O1	0.0130 (4)	0.0173 (4)	0.0219 (5)	−0.0003 (4)	−0.0026 (4)	0.0023 (4)
O2	0.0140 (4)	0.0134 (4)	0.0151 (5)	−0.0015 (3)	0.0037 (3)	0.0017 (3)
O3	0.0251 (5)	0.0138 (4)	0.0179 (5)	0.0040 (4)	0.0086 (4)	0.0022 (4)
O4	0.0138 (4)	0.0332 (6)	0.0169 (5)	−0.0071 (4)	0.0043 (4)	−0.0090 (4)
O5	0.0194 (5)	0.0207 (5)	0.0204 (5)	−0.0044 (4)	−0.0005 (4)	0.0028 (4)
O6	0.0180 (5)	0.0194 (5)	0.0172 (5)	0.0040 (4)	0.0041 (4)	−0.0003 (4)

### Geometric parameters (Å, º)

**Table:**

Ni—O4	2.0226 (11)	P—O3	1.5814 (11)
Ni—O4^i^	2.0227 (11)	P—P^ii^	2.1853 (7)
Ni—O5^i^	2.0544 (11)	O3—H3	0.92 (3)
Ni—O5	2.0544 (11)	O4—H4A	0.82 (3)
Ni—O6	2.0922 (11)	O4—H4B	0.82 (3)
Ni—O6^i^	2.0922 (11)	O5—H5A	0.81 (3)
P—O1	1.5048 (10)	O5—H5B	0.84 (3)
P—O1	1.5048 (10)	O6—H6A	0.81 (2)
P—O2	1.5161 (10)	O6—H6B	0.84 (3)
			
O4—Ni—O4^i^	180.0	O1—P—O3	109.55 (6)
O4—Ni—O5^i^	93.91 (5)	O1—P—O3	109.55 (6)
O4^i^—Ni—O5^i^	86.09 (5)	O2—P—O3	108.73 (6)
O4—Ni—O5	86.09 (5)	O1—P—P^ii^	107.70 (5)
O4^i^—Ni—O5	93.91 (5)	O1—P—P^ii^	107.70 (5)
O5^i^—Ni—O5	179.999 (1)	O2—P—P^ii^	108.69 (5)
O4—Ni—O6	92.97 (5)	O3—P—P^ii^	103.32 (5)
O4^i^—Ni—O6	87.03 (5)	O1—O1—P	0 (10)
O5^i^—Ni—O6	92.80 (5)	P—O3—H3	116.7 (19)
O5—Ni—O6	87.20 (5)	Ni—O4—H4A	119.9 (19)
O4—Ni—O6^i^	87.03 (5)	Ni—O4—H4B	115 (2)
O4^i^—Ni—O6^i^	92.97 (5)	H4A—O4—H4B	109 (3)
O5^i^—Ni—O6^i^	87.20 (5)	Ni—O5—H5A	114 (2)
O5—Ni—O6^i^	92.80 (5)	Ni—O5—H5B	113 (2)
O6—Ni—O6^i^	180.0	H5A—O5—H5B	112 (3)
O1—P—O1	0.00 (11)	Ni—O6—H6A	119.9 (16)
O1—P—O2	117.86 (6)	Ni—O6—H6B	121.2 (19)
O1—P—O2	117.86 (6)	H6A—O6—H6B	111 (2)

Symmetry codes: (i) −*x*+1, −*y*+1, −*z*+1; (ii) −*x*, −*y*, −*z*+1.

### Hydrogen-bond geometry (Å, º)

**Table:**

*D*—H···*A*	*D*—H	H···*A*	*D*···*A*	*D*—H···*A*
O3—H3···O2^iii^	0.92 (3)	1.68 (3)	2.5919 (15)	168 (3)
O4—H4*A*···O3^iv^	0.82 (3)	1.93 (3)	2.7293 (16)	165 (3)
O4—H4*B*···O2^i^	0.82 (3)	1.89 (3)	2.6822 (14)	162 (3)
O5—H5*A*···O1	0.81 (3)	1.98 (3)	2.7770 (15)	171 (3)
O5—H5*B*···O2^v^	0.84 (3)	2.04 (3)	2.8722 (16)	171 (3)
O6—H6*A*···O1^vi^	0.81 (2)	2.05 (2)	2.8163 (16)	159 (2)
O6—H6*B*···O1^iv^	0.84 (3)	2.08 (3)	2.9132 (17)	168 (3)

Symmetry codes: (i) −*x*+1, −*y*+1, −*z*+1; (iii) −*x*, −*y*+1, −*z*+1; (iv) −*x*+1/2, *y*+1/2, −*z*+3/2; (v) *x*+1/2, −*y*+1/2, *z*+1/2; (vi) *x*, *y*+1, *z*.
